# Subjective Experience of Speech Depending on the Acoustic Treatment in an Ordinary Room

**DOI:** 10.3390/ijerph182312274

**Published:** 2021-11-23

**Authors:** Emma Arvidsson, Erling Nilsson, Delphine Bard-Hagberg, Ola J. I. Karlsson

**Affiliations:** 1 Engineering Acoustics, Lund University, John Ericssons väg 1, 223 63 Lund, Sweden; Delphine.Bard@construction.lth.se; 2 Saint-Gobain Ecophon AB, Yttervägen 1, 265 75 Hyllinge, Sweden; erling.nilsson@ecophon.se (E.N.); ola.karlsson@ecophon.se (O.J.I.K.)

**Keywords:** acoustic comfort, acoustic design, room acoustics, sound quality, sound strength, speech clarity, speech perception, reverberation time

## Abstract

In environments such as classrooms and offices, complex tasks are performed. A satisfactory acoustic environment is critical for the performance of such tasks. To ensure a good acoustic environment, the right acoustic treatment must be used. The relation between different room acoustic treatments and how they affect speech perception in these types of rooms is not yet fully understood. In this study, speech perception was evaluated for three different configurations using absorbers and diffusers. Twenty-nine participants reported on their subjective experience of speech in respect of different configurations in different positions in a room. They judged sound quality and attributes related to speech perception. In addition, the jury members ranked the different acoustic environments. The subjective experience was related to the different room acoustic treatments and the room acoustic parameters of speech clarity, reverberation time and sound strength. It was found that people, on average, rated treatments with a high degree of absorption as best. This configuration had the highest speech clarity value and lowest values for reverberation time and sound strength. The perceived sound quality could be correlated to speech clarity, while attributes related to speech perception had the strongest association with reverberation time.

## 1. Introduction

This study deals with the acoustic environment of ordinary public rooms such as classrooms and offices. Typically, complex tasks are performed in these types of environments—tasks that require chains of thought and the processing of information. To accomplish such tasks, appropriate room acoustics are necessary. It is well known that noise can cause stress and disturb concentration [1]. Studies by Kjellberg et al. and Ljung et al. show how the sound environment can also affect cognitive performance [2,3,4]. Those studies show how greater effort is needed to perform tasks if the sound environment is unsatisfactory. Furthermore, the effort required to remember single words is less than that required in order to process information [5]. For non-native listeners, the room acoustic environment is even more critical. Lam [6] could see that greater effort is needed for such listeners to achieve equivalent scores in tests. Furthermore, well-being can also be associated with the acoustic environment [7].

Traditionally, reverberation time, T_20_, is the room acoustic parameter that is controlled in ordinary rooms. This parameter is the time it takes for the sound level to decrease 60 dB from the moment the sound source is turned off. However, it has been found that complementary parameters are needed in order to properly describe the acoustics required to achieve a satisfactory sound environment. One such parameter is speech clarity, C_50,_ accounting for the ratio of early reflections. The early reflections will contribute to the direct sound [8]. Bradley et al. [9] recommend focusing on increasing the ratio of early reflections rather than on lowering the reverberation time in rooms used for speech. It has also been shown in another study by Bradley and Reich that C_50_ can, to some extent, complement a low signal-to-noise (S/N) ratio [10]. Yang and Bradley investigated speech intelligibility for different room acoustic conditions, finding high scores in intelligibility along with an increase in early reflections; S/N also affected speech intelligibility, with a lower effect being seen for varied reverberation time [11]. It has also been seen in a study by Sato et al. that higher energy in early reflections can compensate for a lower sound level with regard to speech intelligibility and listening difficulty [12]. In a study investigating the reading ability of Italian second graders, C_50_ was the best correlating acoustic parameter [13]. C_50_ is included in the standard ISO 3382-1 Acoustics—Measurement of room acoustic parameters—Part 1: Performance spaces [14], where performance spaces are environments such as theatres and concert halls. Another parameter included in this standard and that is normally controlled in performance spaces is sound strength [15,16,17], G, providing information as to how a room responds to the sound source. As mentioned above, T_20_ is normally the focus parameter in ordinary rooms, where the target is often to lower T_20_ by adding absorbing material. The addition of absorbing material will also result in a lower sound level. A risk to be considered in relation to a high degree of absorption is too low a speech level, resulting in not everyone in the audience receiving information [18].

Lately, the need for additional parameters has also been observed in standards, such as the Italian standard UNI 11532 [19], where speech clarity, C_50_, is included for classrooms. In regard to offices, parameters such as the speech transmission index, STI, are included in the standard EN-ISO 3382-3 for acoustic measurements in open offices [20]. Furthermore, a new standard with recommendations for the design of this type of environment has been published: ISO 22955:2021 Acoustics—Acoustic quality of open office spaces [21].

The acoustic properties needed depend on the activity being performed in a room; a classroom with one person speaking places different demands to an open office that could have several sound sources. Typically, several different activities run at the same time in open plan environments. These different environments demand different acoustic properties and, thus, different acoustic treatments.

In studies by Choi [22,23,24], diffusers and absorbers were combined in a small-scale set-up. Using simulations, Labia et al. investigated the placement of absorbers and diffusers in a meeting room environment [25]. In a previous study, a combination of these types of materials was examined in a full-scale mock-up [26]. The results show how an acoustic ceiling is a good baseline, affecting several room acoustic parameters, and also how additional treatment, such as absorbers or diffusers, can be used to fine-tune room acoustics; this means that a combination of these types of solutions can be appropriate.

The placement of the absorbing material is important [27,28]. Azad et al. also investigated the combination of absorbers and diffusers, specifically how a diffuser directing the sound to an absorbent area in a non-diffuse room shows significant effects on the room acoustic parameters evaluated [29]. Shtrepi et al. investigated how the location of diffusers and the distance from them can affect room acoustics, and showed that the perception of room acoustic parameters did not vary significantly with the location of the diffusers, nor were listeners sensitive to the location of the diffusers [30].

The need to consider diffusion and scattering in these types of rooms is also considered in the Italian standard UNI 11532-2 [19] and the German standard 18041 [31].

How diffusers can affect speech intelligibility was investigated by Visentin et al. [32], who identified improvements relating to diffuse reflections as compared to specular. The listening effort related to acoustic design has also been investigated by Visentin et al. [33]. Sanavi et al. studied whether treatment with absorption or diffusing material affects the subjective experience of the acoustics, showing that the jury could recognize both types of treatments, but that the absorber was rated better [34]. In a previous study, evaluation was made of whether people could perceive a difference between various types of acoustic treatments as well as in different positions in the room. It was found that configurations including diffusers were, to a greater extent, perceived as similar in different positions in the room. It was also found that room acoustic parameters such as T_20_, C_50_ and G could not fully explain the subjective experience; other descriptors or the development of measurement techniques could be an alternative. However, C_50_ was the parameter that best explained the subjective experience of the acoustics. A difference of at least 2 dB was needed for the jury to perceive a difference [35]. In a study by Bradley and Reich, it was concluded that 3 dB is a relevant value as a just noticeable difference (JND) in rooms for speech [36]. In ISO 3382-1 [14], in performance spaces, the typical value for JND is 1 dB for C_50_. However, it has been recognized in different studies that the JND can differ for different frequencies and for different types of music [37].

### Study Objective

In ordinary public rooms such as classrooms, complex tasks are performed. From the abovementioned references, it is clear that a room’s acoustics affect cognitive performance. Further, the type of acoustic treatment used will affect the acoustics differently, both objectively and subjectively. The relation between these two aspects, the objective parameters and subjective experience, in ordinary public rooms for speech, is not yet fully understood. The study of this relation has been the objective of the work presented in this article.

The study is part of a research programme aiming at improving the acoustics in ordinary public rooms. In previous studies included in that programme, the effect on room acoustic parameters depending on the type of acoustic treatment was investigated [26]. This was followed up by a study of whether people could subjectively experience a difference between the configurations using different treatments [35]. The subjective experience of sound is further investigated in the study presented in this article by relating the different configurations and the room acoustic parameters to aspects such as sound quality, attributes and ratings of the different environments. This aims at achieving a better understanding of what people prefer when it comes to the acoustics in ordinary public rooms for speech and, in so doing, at increasing the chances of creating satisfactory acoustic environments. The long-term goal is that the outcome from this research can be used in room acoustic design in order to improve people’s acoustic comfort.

## 2. Materials and Methods

In this study, a mock-up area in a laboratory environment was used. The area of the room was approximately 52 m^2^ and the ceiling was installed 2.70 m from the floor. The furnishings were comparable with those of a typical classroom. The room acoustic parameters were measured and sound samples for listening tests were recorded. The following section describes the materials and configurations used in the study, as well as the methods for the measurements and listening tests.

### 2.1. Acoustic Materials

Two different types of acoustic material were used: a sound absorber and a sound reflecting element (a diffuser).

The sound absorber was made of porous material, with a thickness of 40 mm and an air flow resistivity of 40 kPas/m^2^, measured according to ISO 9053-2 [38]. The porous absorber was mounted on the ceiling for all configurations evaluated, and, for one configuration, on the walls. The absorption properties of the material were measured according to ISO 354 [39] for the overall depth of system (ODS) 200 mm and on ODS 50 mm. The ODS value is the distance from the soffit to the top side of the product. 200 mm is the standardised ODS for this type of product. ODS 50 mm can be applied for products mounted directly on the walls. The measured absorption values were evaluated according to ISO 11654 [40], giving a_w_ = 1, which is the highest possible value.

**Figure 1:**
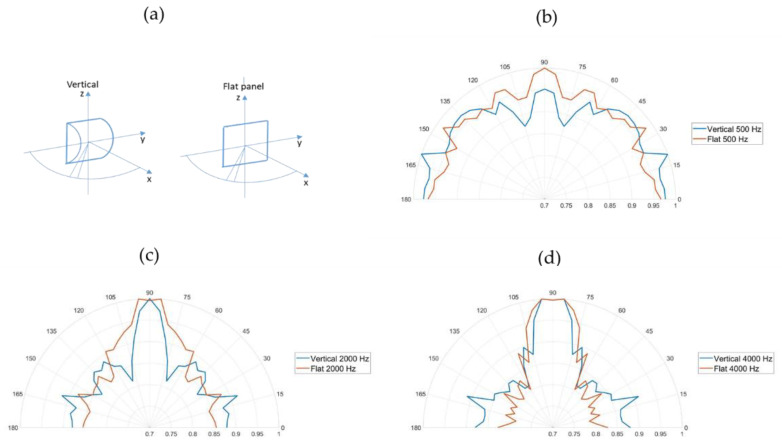
Diffusion characteristics at (**b**) 500 Hz, (**c**) 2000 Hz and (**d**) 4000 Hz. Upper left figure (**a**) shows the orientation of the diffusers’ relative room coordinates.

The diffusing elements had directional diffusing properties in the higher frequency range, but also absorption properties in the lower frequency range, in terms of resonance absorption. The diffusers were mounted in order to direct most of the reflections vertically, i.e., the z-direction. The diffusing characteristics of the diffuser in relation to a flat panel are shown in Figure 1.

### 2.2. Mock-Up and Configurations

Room acoustic measurements and recordings for the listening tests were made in a mock-up with dimensions 7.32 m × 7.57 m × 3.50 m. The room was furnished as a classroom. Eleven tables and eighteen slightly upholstered chairs were used. Two of the tables were used to simulate a teacher’s desk.

**Figure 2:**
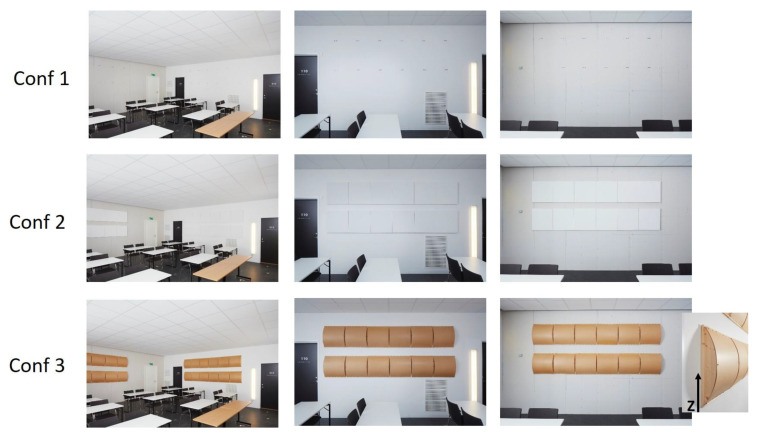
Configurations used in the experiments. Upper row: Conf. 1. 52 m^2^ absorbent ceiling. The room was furnished. Second row: Conf. 2. 52 m^2^ absorbent ceiling; 9 m^2^ absorbing tiles distributed on two walls. The room was furnished. Third row: Conf. 3. 52 m^2^ absorbent ceiling; 9 m^2^ diffusers distributed on two walls. The room was furnished.

Three different room configurations were investigated. In all configurations, an absorbent ceiling was used, installed at a height of 2.70 m from the floor. In configuration 1, no other treatment than the ceiling was used. In configuration 2, porous absorbers were mounted on two perpendicular walls. In configuration 3, diffusers replaced the absorbers on the walls. The diffusers redirected the majority of sound waves in vertical direction. The three configurations are presented in Figure 2.

### 2.3. Room Acoustic Measurements

**Figure 3:**
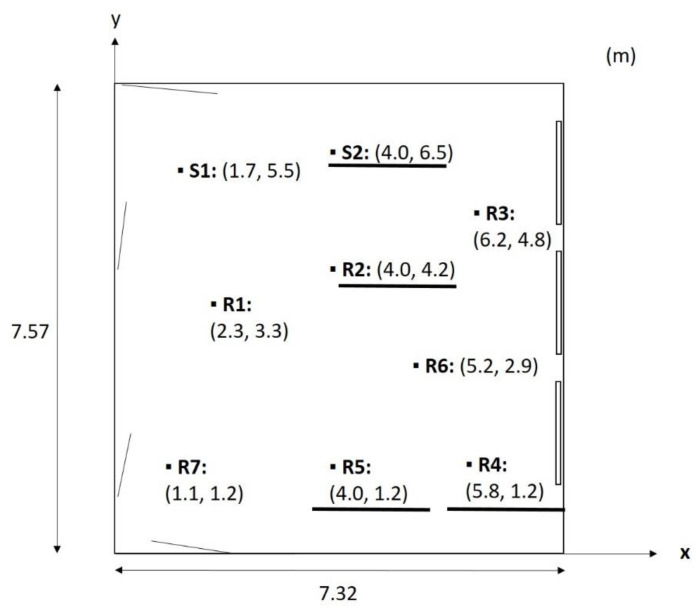
Source and receiver positions. Two source positions: S1 and S2. Seven receiver positions: R1–R7. All positions were used in room acoustic measurements. In sound sampling for listening tests, source S2 and receiver positions R2, R4 and R5 were used, underlined in the figure.

The room acoustic parameters evaluated were reverberation time (T_20_), speech clarity (C_50_) (Equation (1)) and sound strength (G) (Equation (2)). Two source positions and seven receiver positions were used—see Figure 3, where ‘R’ indicates receiver and ‘S’ indicates source. Details of the measurement procedure of these measurements can be found in our previously published article [35].

Speech clarity, C_50_, is defined as:

(1) $$C_{50}=10lg\frac{\int_{0}^{50\mathrm{ms}}h^{2}\left(t\right)dt}{\int_{50\mathrm{ms}}^{\infty }h^{2}\left(t\right)dt}\;\left[\mathrm{dB}\right]$$

Sound strength, G, is defined as:

(2) $$G=10lg\frac{\int_{0}^{\infty }h^{2}\left(t\right)dt}{\int_{0\mathrm{ms}}^{t_{dir}}h_{10m}^{2}\left(t\right)dt}\;[\mathrm{dB}]$$

where

h(t) is the impulse response.

h_10m_ is the impulse response at 10 m in a free field.

For both speech clarity and sound strength, the early reflections are included. When evaluating T_20_, according to ISO 3382-2 [41], the evaluation interval is −5 dB to −25 dB, given that the early reflections are excluded.

The measurements were performed during the course of one day with stable temperature and humidity conditions. The background noise was <30 dBA.

### 2.4. Sound Sampling and Listening Test Design

Sounds for the listening tests were sampled by recording sounds in the same environment on the same day as the room acoustic measurements were performed. Female speech, sampled in an anechoic chamber, was played from a loudspeaker, type Genelec 8030B placed in S2, with the acoustic centre at a height of 1.55 m from the floor. The emitted sound power level was the same for all samplings.

**Figure 4:**
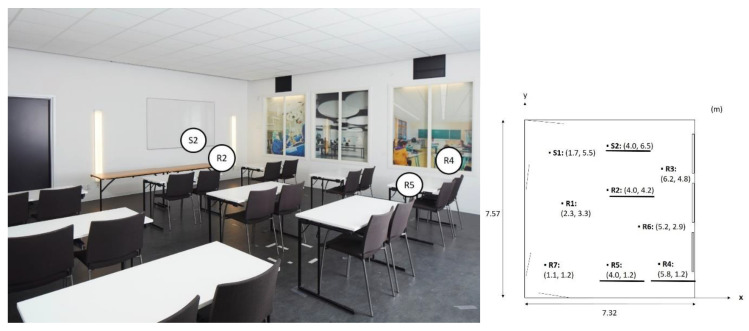
The positions used for sound sampling. The speech was emitted from S2 and sampled in R2, R4 and R5. In the sketch to the right, the positions used in sound sampling are underlined. All positions shown in the sketch were used for the averaged values of room acoustic measurements.

**Figure 5:**
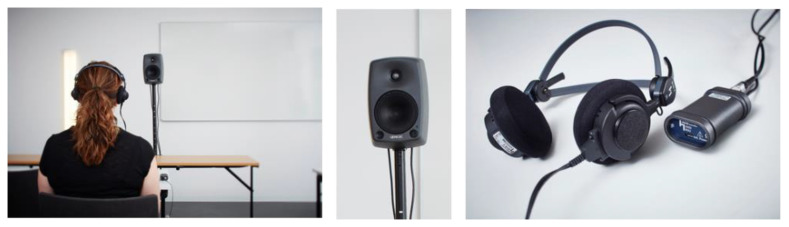
Set-up for sound sampling. Loudspeaker, Genelec 8030B, used as sound source placed in position S2. The right picture shows headphones BHS II and adapter B2U for recording the sounds and equalisation for listening test. Sampling was conducted in R2, R4 and R5.

Recordings were made with binaural headphones, type B2U, HEAD acoustics. Each sample was 4–6 s long and was recorded at height of 1.20 m from the floor. Recordings were made with binaural headphones, BHS II (3322) HEAD Acoustics (HEAD acoustics GmbH: Herzogenrath, Germany), with calibrated microphones. A B2U (3323) HEAD Acoustics adapter (HEAD acoustics GmbH: Herzogenrath, Germany), was used for recording and playback equalisation and the same headphones were used for recordings and playback during listening tests. Recordings were made in positions R2, R4 and R5. The different positions were the same as described in the previous section on room acoustic measurements; however, the chosen positions for the listening test can be seen in Figure 4. The set-up for sound samplings is shown in Figure 5.

Due to the challenge of creating a test including people’s subjective experience, a number of aspects had to be considered in the test design. One such aspect was the duration of the test in relation to the ability of the jury members to maintain concentration during the test session. The test was designed to last a maximum of 20 min. To avoid bias for any of the participants, the test was conducted in a neutral room. In addition, none of the jury members had any involvement in the study, and the sounds were encoded. This meant that no information about the different sounds could be connected to a specific position or configuration for any of the participants. Another aspect that may affect the outcome of a perception test is personal status, such as medical health and mood. Self-reporting on these aspects was included in the test session. The participants rated their mood at the specific time of the performance of the listening test. The scale for this rating was 0–5, with 0 meaning not good at all and 5 meaning excellent. Furthermore, the question of ability to concentrate was reported after the test. A scale was also used here, from 0–10. With regard to concentration, 10 was deemed to be not difficult to concentrate. Concerning instructions, 10 indicated that the instructions were totally clear. The responses to these questions, i.e., regarding mood, health, ability to concentrate, etc., could be considered if any outlier results were found.

Before the test was conducted, a pilot study was performed. Five people did the test, which was followed up with a discussion of the test itself. The outcome from the pilot study led to clarifications in the instructions and shorter sound samples. Consequently, the results from the pilot study are excluded from the analysis and results presented in the coming sections.

The jury consisted of a randomised sample of 29 people. Fifteen of the participants were female. In the first part of the test, a training session was performed. This training gave instructions on how to use the software, the different types of tests were demonstrated and necessary definitions were explained. Additionally, participants were asked to consider themselves to be in a classroom where they were listening to information and were encouraged to make their choices based on their first impressions.

The perception test included three different types of judgements: judgement of sound quality, choosing pre-defined attributes and ranking. The judgements were applied for each of the three configurations in the three different receiver positions, i.e., nine points were considered in total.

Regarding sound quality, a scale with 10 grades was used. Number 1 corresponded to intolerable and 10 to excellent. People were asked to consider themselves sitting in a room where they listen to information when rating on the 10-point scale.

Mean and median values were evaluated and related to the different configurations. The 10-point scale for sound quality was also subdivided into three groups in order to obtain an understanding of the overall impression of quality for the different configurations and the room acoustics. The following sound quality groups were used:

A: Satisfactory, corresponding to points 8–10

B: Acceptable, corresponding to points 5–7

C: Unsatisfactory, corresponding to points 1–4.

Predefined attributes were related to speech perception. The jury participants were not acousticians, and the attributes were described for the participants in general terms, as follows:

Echoic- Echo/tendencies of echo.

Unclear- No echo, but indistinct; you need to concentrate extra hard to hear.

Clear- The sound is clear but not comfortable to listen to.

Pleasant- The sound is clear and comfortable to listen to.

With regard to the results on sound quality as well as the attributes, correlation and regression were investigated. The sound quality or attributes were, in these cases, set as the response variable, and room acoustic parameters were the exploratory variable.

Regarding correlation, it was investigated whether a linear association existed and, in addition, the strength and statistical significance of any such correlation. The value ‘r’ indicates the quality of the correlation, in the range −1 ≤ r ≥ 1. r = 0 means no correlation, while r = 1 and r = −1 mean total correlation. In the evaluation of statistical significance, the confidence interval was set to 95%. Using the Null Hypothesis (H_0_) set to ρ = 0, no linear relationship existed between the variables. The Alternative Hypothesis (H_1_) was set to ρ ≠ 0; a linear relationship did exist. The significance level α = 0.05. The hypothesis was tested using the *p*-value associated with the correlation coefficient. If *p* ≤ α, we reject H_0_; if *p* > α, we fail to reject H_0_.

Minitab^®^ 19.1.1 was used for the statistical analysis.

For the regression, one explanatory variable was used, meaning simple regression was applied. The degree of explanation (R^2^) was evaluated.

In the ranking session, the participant was asked to rank which sound out of three they preferred if they were listening to information in a classroom. Sounds from the three different configurations in the same position were used in this session, i.e., the rating was relative to the different configurations.

## 3. Results

This section presents the results on room acoustic parameters in Section 3.1, followed by the results on subjective experience in Section 3.2.

### 3.1. Room Acoustic Parameters

The three different room acoustic parameters of reverberation time (T_20_), speech clarity (C_50_) and sound strength (G) were evaluated in octaves from 125–4000 Hz. The average values over the two source positions and seven receiver positions (see Figure 3) were evaluated. Furthermore, the specific positions R2, R4 and R5, i.e., the positions for which listing tests were performed, were evaluated. For these specific positions, sound source position S2 was used, i.e., the same source position used for emitting speech in the listening tests.

Starting with the average values, the configuration without wall treatment (conf. 1) gave the highest T_20_ values throughout the full frequency range evaluated. With regard to the configurations with wall treatment, the values of T_20_ were slightly lower at frequencies 500–4000 Hz for the configuration with porous absorbers on the wall (conf. 2). However, in the lowest frequency range evaluated, 125–250 Hz, the T_20_ was lowest for the configuration with diffusing elements (conf. 3). These lower values are due to resonance absorption in the diffusing elements.

The previously discussed results concerning T_20_ are valid both for the average values and for the specific positions.

With regard to C_50_, configuration 1 gave the lowest values, i.e., the highest number of late reflections considered to disturb the speech clarity, throughout the full frequency range evaluated. This is valid in relation to both the average and specific position values.

Configuration 2 had, on average, the highest values in C_50_, i.e., the highest number of early reflections contributing to improved speech clarity, at frequencies 500–4000 Hz. A slightly higher value in C_50_ was seen for configuration 3 in the low frequency range of 125–250 Hz. This means that the same trends regarding frequency range were seen for T_20_ and C_50_.

The evaluation of sound strength showed the lowest values for the configuration with the highest amount of absorption, configure 2. Configurations one and three had similar values in G. It should be noted that, when considering the average values, only small variations were noted for this parameter, as all configurations had an absorptive ceiling that significantly regulated this parameter.

The specific receiver positions R2, R4 and R5 were evaluated, with S2 as source position. The results in these positions were the ones used in comparison with the responses in the listening test. Regarding the reverberation time, T_20_, the trends for the different positions were similar to the average values. At frequencies from 500 Hz and upwards, the lowest value in T_20_ was seen for configuration 2, containing the most absorption. The resonance absorption properties of the diffusers were also seen in the different positions, with configuration 3 giving the lowest T_20_ values at low frequencies.

There was considerable variation in the parameter C_50_, depending on position. Differences of 3 dB or more were seen for all configurations when the three specific positions were compared. The difference, depending on treatment, in the positions in the rear part of the room should be noted. In the higher frequency range, C_50_ was lower for configuration 2 than for configuration 3 in position R5. This is the opposite compared to the average values, indicating the importance of also considering the specific position in a room. The lowest values in C_50_ were obtained for configuration 1, in all specific positions, compared to the average values.

Sound energy was reduced by the distance from the source and, in positions further away from the speaker, positions R4 and R5, the values of G were more than 1 dB lower at frequencies 2000–4000 Hz for configuration 2. Smaller differences were seen for configurations 2 and 3.

The mechanism causing the changes in room acoustic parameters for configurations 2 and 3 differ. With regard to configuration 2, the sound energy is absorbed and thus the acoustic parameters are adjusted. For configuration 3, the sound waves are broken up by the diffusing elements in different directions. The soundwaves are thus prevented from travelling back and forth between the walls. The fact that sound waves can travel back and forth between the walls is the reason for the highest T_20_ and lowest C_50_ for configuration 1. The adjustments of the reflection pattern with diffusers alter T_20_ and C_50_, while the sound energy in the room remains. Thus, the sound strength, G, did not change for configuration 3 in relation to configuration 1 in the high frequency range. With the use of absorbers, G is also affected. Slightly lower G at low frequencies for configuration 3 is due to the resonance absorption by the diffusers.

Graphs with the results on room acoustic parameters, average and specific positions can be found in Supplementary S1.

### 3.2. Listening Test

In the following sections, the results from the listening test are presented. It should be noted that the participants in the listening test had no insight into the study, the tests were performed in a neutral room and all sounds were decoded. This means that the jury members did not have any information about the different sounds they were listening to.

#### 3.2.1. Jury Members

In order to identify any potential differences in the responses, an ANOVA was used for the evaluation of sound quality and attributes.

Regarding sound quality, no specific outlier was found. Seven jury members responded with slightly lower values. In the group of seven who answered with lower values, all found the instructions to be totally clear. Their ages varied from 26 to 48 years. Two members found it more difficult to concentrate during the full test, one reported a bad mood and one reported having a hearing aid. This means that no common attribute for the seven members could be found. Consequently, none of these seven responses were removed in the evaluation of sound quality.

One jury member answered more often with high quality values for the configuration with no additional wall treatment and with diffusers. However, this person still followed the same trend as the mean values. Mean and median values did not change on removal of this person’s responses. These responses were thus also used in the evaluation of the sound quality.

With regard to outliers, no outlier could be identified in the ANOVA made for the attributes on speech perception. However, there was a trend of three jury members giving higher ratings, i.e., more often choosing clear or pleasant. These three members reported that they could maintain concentration, that the instructions were clear and that they were in a good mood. No hearing aids were reported for these three members. However, one of them was the same person who reported high sound quality values. As no significant outlier results were found, and as individual preferences are natural, all responses were used in the evaluation of the attributes.

#### 3.2.2. Sound Quality

The sound quality was rated on a 10-point scale, where 1 was described as intolerable and 10 as excellent. The jury members were asked to consider that they were in a situation listening to information. All 29 jury members judged the sound quality for the three positions R2, R4 and R5 for three configurations (see Figure 4).

For all configurations, position R2, i.e., the position closest to the speaker, was judged to have the highest sound quality when considering each configuration.

Configurations 2 and 3 were judged to have the same value for this position, a quality of 9, in both mean and median values. No jury member rated this position lower than 7, which was considered to be good sound quality. Regarding position R4, configurations 1 and 3 were judged to be the same when mean and median values were considered, with a result of 6 in sound quality, which was still considered good. For configuration 2, the same position, R4, was judged as a 7 in mean and median. This configuration was judged to have the same quality in position R5, i.e., a sound quality of 7, while configuration 3 got 6 and configuration 1 got 5 when the mean values were considered.

Overall, configuration 2 was judged to be the best when this quality scale was used. Moreover, Q1 had the lowest value of 5 for position R5, which was still considered acceptable. However, for configurations 1 and 3, Q1 had a value of 4, which was not satisfactory. It should also be mentioned that the variations between the 29 jury members were lowest for configuration 2. The descriptive statistics concerning the sound quality are shown in Table 1.

The sound quality reported by the jury members was divided into different groups in order to obtain an understanding of which configurations could be deemed satisfactory or not. Three different groups were created:

A. Satisfactory: sound quality level 8–10.
B. Acceptable: sound quality level 5–7.
C. Unsatisfactory: sound quality level 1–4.

**Figure 6:**
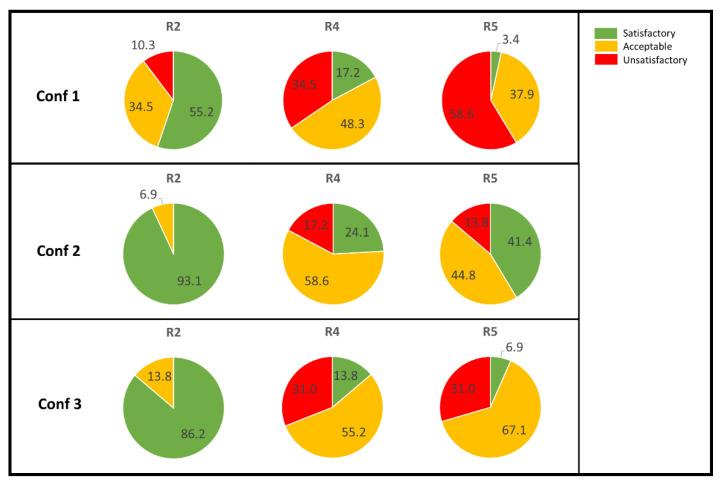
Sound quality grouped into three levels, A: satisfactory (green), B: acceptable (yellow), and C: unsatisfactory (red). The results for the position closest to the speaker, R2, are seen in the first column; the results for the position in the corner, R4, are seen in the middle column; and the results for the position in the back, R5, at the same distance in x as the speaker position, are seen in the right column.

When the data from all the individual judgements were evaluated, it was found that 10% of the jury members judged configuration 1, position R2, to be unsatisfactory. Concerning the same position for configurations 2 and 3, the majority were in group A: satisfactory. For position R4, in the corner of the room, some jury members judged the sound quality at levels corresponding to group C: unsatisfactory; this was obtained for all configurations, but in different positions. For configuration 1, as many as 35% judged it unsatisfactory. For configurations 2 and 3, 17% and 31% judged it unsatisfactory, respectively. For position R5 in configuration 1, the majority, 59%, judged the quality at levels corresponding to unsatisfactory. The same position, R5, for configuration 3 was also judged to be unsatisfactory by 31% of the jury members. Only 13% of the responses were in the unsatisfactory range for configuration 2 in position R5. The results for the grouping are shown in Figure 6.

It was investigated whether any association between the responses in sound quality and the room acoustic parameters exists. The mean values of the responses from the jury members for each listening position, R2, R4 and R5, for the three configurations were used. This means that a total of nine points were evaluated. In respect of the room acoustic parameters, the values of T_20_, C_50_ and G for octaves 125–4000 Hz for the same positions and configurations were used.

The Pearson correlation gave responses on correlation (r) and *p*-values. Regarding the association between sound quality and T_20_, the response in correlation quality was low. The highest value was seen for a frequency of 500 Hz, with r = −0.664. The *p*-value was 0.051, meaning statistical significance was just on the borderline.

For C_50_, a higher correlation was obtained, especially for the higher frequency range of 1000 Hz–4000 Hz. The correlation coefficient for C_50_ at 1000 Hz was 0.824, for 2000 Hz 0.921 and for 4000 Hz 0.817. These r-values can be interpreted as a good correlation for a subjective test like this listening test. In addition, the *p*-values showed a statistical significance (*p* < 0.05) for these frequencies. In the regression investigation, C_50_ was used as the explanatory value. For the frequency range with high correlation quality, i.e., 1000–4000 Hz, a linear regression with good R^2^ was found. For 1000 Hz, the R^2^ was 68%, for 2000 Hz 85% and for 4000 Hz 67%. At the lower frequencies, low correlation quality was obtained. A regression analysis was also made, but resulted in a low R^2^. With regard to G, no association with sound quality could be found. It should be observed that low variations in G were obtained between the positions and configurations investigated. The correlations for the sound quality and room acoustic parameters are presented in Table 2. Equations for the linear regression, together with its degree of explanation, can be found in Supplementary S2.

#### 3.2.3. Attributes

The responses from all 29 members of the jury are presented below.

Evaluation of the attributes for all 29 participants shows high scores of echo in the configuration where no acoustic material was used on the walls (conf. 1). In the position close to the speaker, 66% deemed it echoic, and the position on the line from the source but at the back of the room, R5, was deemed to be echoic by 80% of the jury members. Sixty-nine per cent judged the position at the back corner, R4, to be echoic.

With regard to the configurations with absorption on the walls, the majority found the sound to be clear or pleasant in all positions. However, 17% found the position in the back corner, R4, to be unclear.

**Figure 7:**
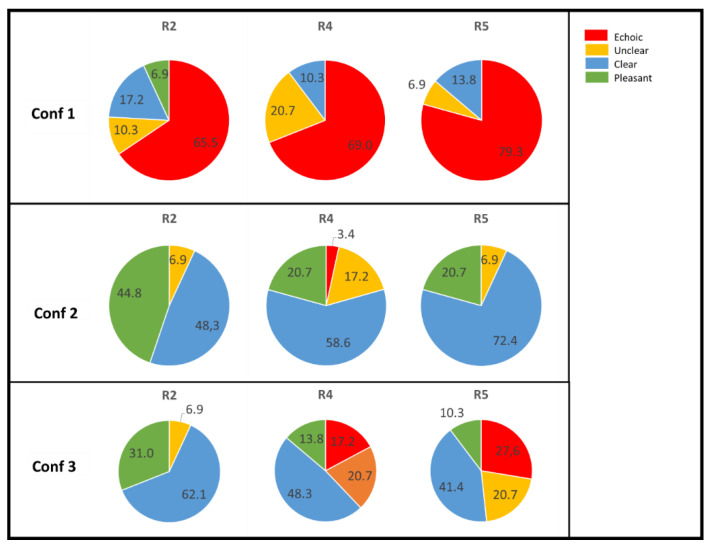
Pie chart for the 29 participants’ judgement of attributes. 1/red = echoic, 2/orange = unclear, 3/yellow = clear, 4/green = pleasant. The results for the position closest to the speaker, R2, are seen in the first column; the results for the position in the corner, R4, are seen in the middle column; and the results for the position in the back, R5, at the same distance in x as the speaker position, are seen in the right column.

For the configurations with diffusers on the walls, the majority judged the acoustic environment to be clear or pleasant. For the position close to the speaker, position R2, >90% deemed it to be clear or pleasant. However, for the positions at the back, a considerable number of jury members judged the environment to be unclear or even echoic. Twenty-eight per cent found the position on the line from the speaker at the back, R5, to be echoic. For the position in the back corner, R4, 17% found it echoic. A pie chart presents the results of jury members’ perception of the attributes (Figure 7).

A correlation analysis was performed using the Pearson correlation. The association between the attributes and room acoustic parameters T_20_, C_50_ and G over the octaves 125–4000 Hz was investigated. The attributes are explained by number, where 1 corresponds to echoic, 2 corresponds to unclear, 3 corresponds to clear and 4 corresponds to pleasant. The mean value of the 29 participants’ judgements was used in the analysis.

The highest strength of correlation to the attributes was found for T_20_ at frequencies 500–4000 Hz. The correlation quality for T_20_ at 500 Hz was r = −0.877, for 1000 Hz r = −0.915, for 2000 Hz r = −0.90 and for 4000 Hz r = −0.889. For these frequencies, the *p*-value was <0.05, meaning the correlation was statistically significant. Linear regression for T_20_ at these frequencies was found to get an R^2^ range from 77 to 84%, with the highest value being obtained for 1000 Hz.

Regarding C_50_, a fairly high correlation strength was found in the higher frequency range, 1000–4000 Hz, but not as high as for T_20_. For C_50_ at 1000 Hz, r = 0.810, at 2000 Hz r = 0.708 and at 4000 Hz r = 0.708. At these frequencies, the *p*-value was <0.05, meaning statistical significance was obtained. Linear regression analysis showed R^2^ ranging from 50% to 66%.

Regarding G, a low correlation of quality with attributes was obtained. As mentioned earlier, the variations for this parameter were low between the positions and configurations investigated. The correlation data are presented in Table 3. The equations for linear regression, together with the degree of explanation, can be found in Supplementary S2.

#### 3.2.4. Ranking

In the ranking part of the listening test, the participants answered in respect of which configuration they would prefer if sitting in an environment listening to information. The ranking was made for comparison between the configurations, for each position, i.e., positions R2, R4 and R5.

The results show the same ranking for all positions. Configuration 2 was rated as number one, configuration 3 as number two and configuration 1 as number three. However, the responses were differently distributed for the different positions. In position R5, configuration 2 was rated most frequently as the preferred option, with the lowest deviation for the three different positions. In position R4, configuration 2 was just slightly better than configuration 3. Configuration 1 was clearly rated as number three for all positions. The results are found in Table 4.

## 4. Discussion

Three different configurations were investigated from objective and subjective perspectives. From an objective perspective, the three room acoustic parameters of reverberation time (T_20_), speech clarity (C_50_) and sound strength (G) were measured. The average values as well as specific receiver positions were considered. With regard to the different receiver positions, a large difference was observed between some positions. This stresses the importance of considering the acoustic properties in different locations of the room, even for ordinary rooms. In the acoustic design of performance spaces, such as theatres and concert halls, the typical procedure is to make sure everyone in the audience enjoys a good acoustic experience. Ensuring good listening conditions for everyone in the audience in ordinary rooms, such as classrooms, should be just as natural.

With regard to the listening test, all participant responses were used in the evaluation, as no outliers could be identified. The fact that differences in the jury members’ mood or ability to concentrate did not affect the results may be due to the results found being independent of these types of aspects.

With regard to the correlation and regression analysis, a strong correlation to the room acoustic parameters could be found, mainly in the higher frequency range. This can be explained by the fact that the acoustic treatment used operated mainly in that frequency range. Additionally, due to the greater sensitivity of our hearing at the higher frequencies and because speech normally contains mainly high frequency sounds, the sounds that the jury judged were in this higher frequency range.

The diffusers contributed low frequency absorption at 125–250 Hz. This difference seems not to have affected people’s judgement for any of the perception evaluations. However, it should be kept in mind that female speech was used in the study. If male speech had been used, more low frequency sound could have been emitted, resulting in the low frequency absorption playing a more significant role and thus leading to other responses in the perception tests.

The study included a rating of the different configurations. In addition, the sound quality judgements can be used in analysing preferred solutions. These two evaluations show the same results: the preferred configuration in ranking and the configuration with the best sound quality was the configuration with an absorbent ceiling and absorbers on the walls (conf. 2). This solution has the lowest reverberation time (T_20_), the highest speech clarity (C_50_) and the lowest sound strength (G).

Whether it was the type of treatment or the values of the room acoustic parameters that people prefer was also considered by studying the correlation between people’s subjective experience and the different room acoustic parameters investigated, resulting in some indications.

The correlation found between sound quality and C_50_ for the frequency range 1000–4000 Hz can be regarded as strong, with a correlation quality of 0.817–0.921. The *p*-values showed statistical significance and, in addition, a high degree of explanation could be seen for the regression using C_50_ for these frequencies as explanatory variables. The other room acoustic parameters measured (T_20_ and G) did not show any correlation, giving strength to a cause and effect relation existing between speech clarity and perceived quality for frequencies 1000–4000 Hz. We could thereby assume that C_50_ does explain the cause and effect for perceived sound quality.

Regarding the correlation between the attributes, the strongest correlation was found to reverberation time (T_20_). The r-value showed a strong correlation, with r-values from −0.877 to −0.915, and *p*-values showing statistical significance for this correlation. However, for C_50_ as well, a correlation to the attributes in the higher frequency range was seen. This correlation was not as strong as for T_20_, indicating that T_20_ does not fully explain the effect of perception on the attributes used in this study.

No correlation to G was observed. This may depend on the fact that the acoustic configurations used resulted in small variations in G, and this parameter had already been adjusted by an acoustic ceiling. However, other types of questions to the jury could be better associated to this parameter, such as regarding loudness.

From the results on the correlation between room acoustic parameters and people’s subjective experience, it seems that C_50_ is a good descriptor for people’s perception of speech. Sound quality evaluation indicates that a C_50_ > 8 dB was needed to obtain satisfactory sound quality. C_50_ can be adjusted either by adding diffusers or absorbers, with higher values being obtained with the absorbers.

In a previous study [40] in this research programme, it was found that the configuration with diffusers on the walls, configuration 3, gave a more similar subjective experience than the one with absorbers. The fact that people have the same possibilities to hear speech independent of the position in the room is, of course, of great importance. The combination of these findings can be useful in the future acoustic design of ordinary rooms.

## 5. Conclusions

This study investigated how people experience speech in three different acoustic environments. Twenty-nine people judged speech that was recorded with binaural headphones in different positions in the different environments. The observations from people were related to different types of acoustic treatments as well as to different room acoustic parameters. From this evaluation, it can be concluded that it is important to consider C_50_ in rooms for speech, as C_50_ effectively describes people’s perception of speech and is, in addition, related to their experience of sound quality. To obtain satisfactory sound quality, C_50_ > 8 dB is required. The achievement of this value should be controlled in several positions in the room and not only on an average basis. In order to achieve a C_50_ of 8 dB, an absorbent ceiling is a good baseline, but additional treatment, either absorbing or diffusing, is needed. However, from previous studies, it has been found that diffusers contributed to a more uniform experience of sound.

## Figures and Tables

**Table 1:** Descriptive statistics of sound quality judgements.

Variable	N	Mean	St. Dev	Variance	Minimum	Q1	Median	Q3
Conf. 1_R2	29	7	2.0	3.8	3	7	8	9
Conf. 1_R4	29	6	1.9	3.8	2	4	6	7
Conf. 1_R5	29	5	1.6	2.5	2	4	4	6
Conf. 2_R2	29	9	0.8	0.7	7	9	9	10
Conf. 2_R4	29	7	1.7	2.7	3	7	7	8
Conf. 2_R5	29	7	1.6	2.5	4	5	7	8
Conf. 3_R2	29	9	0.9	0.8	7	8	9	9
Conf. 3_R4	29	6	1.9	3.7	2	4	6	7
Conf. 3_R5	29	6	1.7	2.8	3	4	6	7

**Table 2:** Results from correlation and regression analysis for perceived sound quality and room acoustic parameters. A good correlation between C_50_, in the higher frequency range, can be noted. Results with *p* < 0.05 are marked in bold.

Room Acoustic Parameter	Frequency (Hz)	Pearson Correlation Sound Quality—Room Acoustic Parameter 95% CI	
		Quality (r)	*p*
T_20_	125	−0.015	0.969
T_20_	250	−0.334	0.380
T_20_	500	−0.664	0.051
T_20_	1000	−0.588	0.096
T_20_	2000	−0.543	0.131
T_20_	4000	−0.583	0.099
C_50_	125	0.527	0.145
C_50_	250	0.546	0.129
C_50_	500	0.492	0.179
**C_50_**	**1000**	**0.824**	**0.006**
**C_50_**	**2000**	**0.921**	**0.000**
**C_50_**	**4000**	**0.817**	**0.007**
G	125	0.258	0.503
G	250	−0.524	0.148
G	500	−0.072	0.853
G	1000	0.158	0.685
G	2000	0.299	0.434
G	4000	0.336	0.377

**Table 3:** Results from analysis on correlation and regression for attributes and room acoustic parameters. The best correlation was found for T_20_. However, C_50_ also gave fairly good correlation in the higher frequency range, meaning that T_20_ cannot fully explain the attributes. Results with *p* < 0.05 are marked in bold.

Room Acoustic Parameter	Frequency (Hz)	Pearson Correlation Attributes—Room Acoustic Parameter 95% CI	
		**Quality (r)**	***p***
T_20_	125	−0.325	0.393
T_20_	250	−0.509	0.162
**T_20_**	**500**	**−0.877**	**0.002**
**T_20_**	**1000**	**−0.915**	**0.001**
**T_20_**	**2000**	**−0.907**	**0.001**
**T_20_**	**4000**	**−0.889**	**0.001**
C_50_	125	0.247	0.522
C_50_	250	0.169	0.663
C_50_	500	0.543	0.131
**C_50_**	**1000**	**0.810**	**0.008**
**C_50_**	**2000**	**0.708**	**0.033**
**C_50_**	**4000**	**0.767**	**0.016**
G	125	0.373	0.322
G	250	0.659	0.054
G	500	−0.394	0.295
G	1000	0.040	0.918
G	2000	0.088	0.823
G	4000	0.074	0.850

**Table 4:** Results on rating for sound in different positions for the different configurations.

	Conf. 1_R2	Conf. 2_R2	Conf. 3_R2	Conf. 1_R4	Conf. 2_R4	Conf. 3_R4	Conf. 1_R5	Conf. 2_R5	Conf. 3_R5
Average	2.86	1.17	1.97	2.72	1.45	1.83	2.86	1.10	2.03
Standard deviation	0.34	0.46	0.49	0.52	0.72	0.59	0.51	0.40	0.18
Confidence (0.95)	0.13	0.17	0.18	0.19	0.26	0.22	0.18	0.15	0.07
Lower Quartile	3.00	1.00	2.00	3.00	1.00	1.00	3.00	1.00	2.00
Median	3.00	1.00	2.00	3.00	1.00	2.00	3.00	1.00	2.00
Upper Quartile	3.00	1.00	2.00	3.00	2.00	2.00	3.00	1.00	2.00

## Data Availability

The data presented in this study are available on request from the corresponding author.

## Supplementary Materials

The following are available online at https://www.mdpi.com/article/10.3390/ijerph182312274/s1, Figure S1. Average values over two sources and seven receiver positions for room acoustic parameters reverberation time, T20, speech clarity, C50, and sound strength, G; Figure S2. The three room acoustic parameters evaluated, reverberation time, T20, speech clarity, C50, and sound strength, G, for specific receiver positions using source position S2 and receiver position R2, i.e., close to the speaker; Figure S3. The three room acoustic parameters evaluated, reverberation time, T20, speech clarity, C50, and sound strength, G, for specific receiver positions using source position S2 and receiver position R4, i.e., in the corner.; Figure S4. The three room acoustic parameters evaluated, reverberation time, T20, speech clarity, C50, and sound strength, G, for specific receiver positions using source position S2 and receiver position R5, i.e., same x-position as the speaker in the rear part of the room. Table S1. Configuration Description; Table S2. Equations for linear regression with degree of explanation, R2, for sound quality using room acoustic pa-rameters as explanatory parameter; Table S3. Equations for linear regression with degree of explanation, R2, for attributes using room acoustic parameters as explanatory variable.
