# Supplementary material for: Subjective Experience of Speech Depending on the Acoustic Treatment in an Ordinary Room
Source: Int J Environ Res Public Health. 2021 Nov 23;18(23):12274. doi: 10.3390/ijerph182312274 (PMC8657266; doi:10.3390/ijerph182312274)
Supplement: Supplementary file 1 [file ijerph-18-12274-s001.zip › ijerph-1454273- Supplementary 1.pdf]

International Journal of
Environmental Research and Public Health

Supplementary 1: Results on Room Acoustic Parameters

Following four graphs presents the results on room acoustic parameters. **Table S1** below explains the set-up for each configuration.

Table S1. Configuration Description.

Configuration		Ceiling	Wall treatment
**No**	**Abbreviation**		
1	52CA_F	52 m^2 absorptive	No wall treatment
2	52CA_9WA_F	52 m^2 absorptive	9 m^2 absorptive tiles distributed on two walls
3	52CA_9VWD_F	52 m^2 absorptive	9 m^2 vertically oriented diffusers distributed on two walls

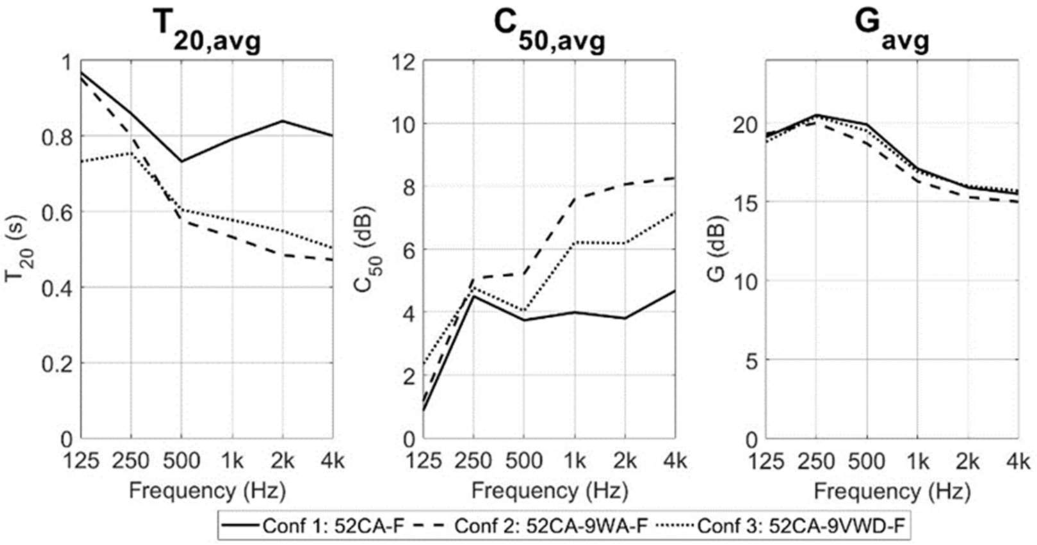

Figure S1. Average values over two sources and seven receiver positions for room acoustic parameters reverberation time, T20, speech clarity, C50, and sound strength, G.

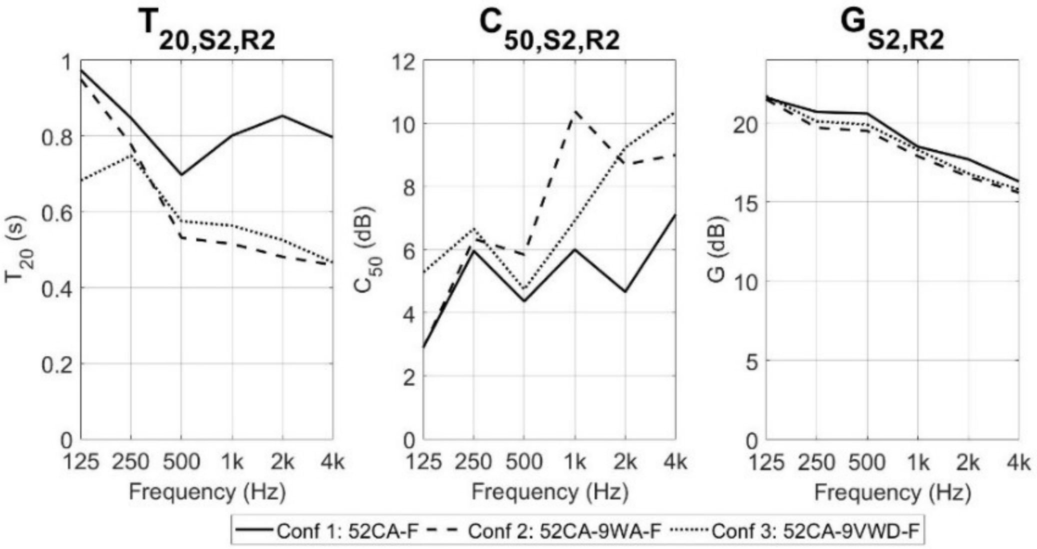

Figure S2. The three room acoustic parameters evaluated, reverberation time, T_{20}, speech clarity, C_{50}, and sound strength, G, for **specific receiver positions** using source position S2 and **receiver position R2**, i.e., close to the speaker.

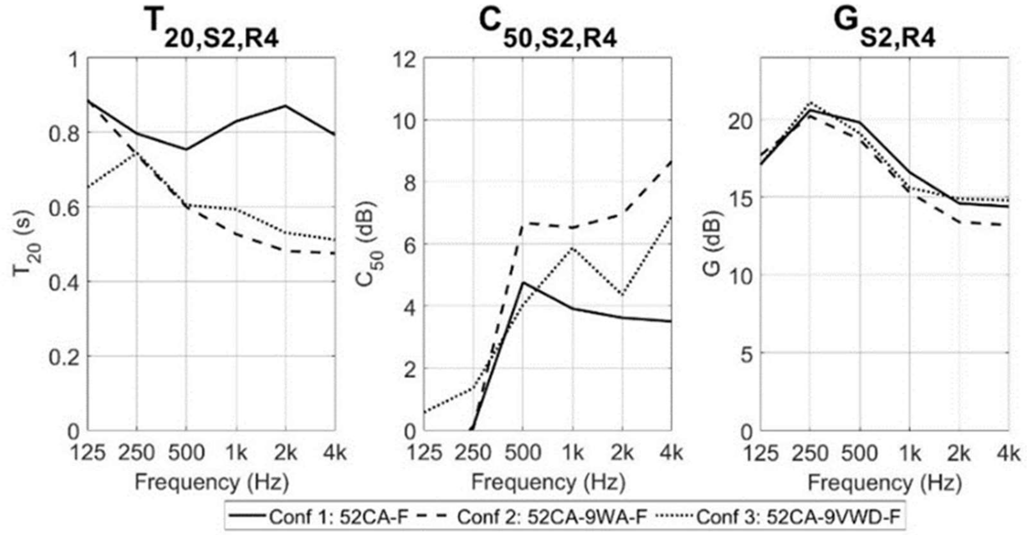

Figure S3. The three room acoustic parameters evaluated, reverberation time, T_{20}, speech clarity, C_{50}, and sound strength, G, for **specific receiver positions** using source position S2 and receiver **position R4**, i.e., in the corner.

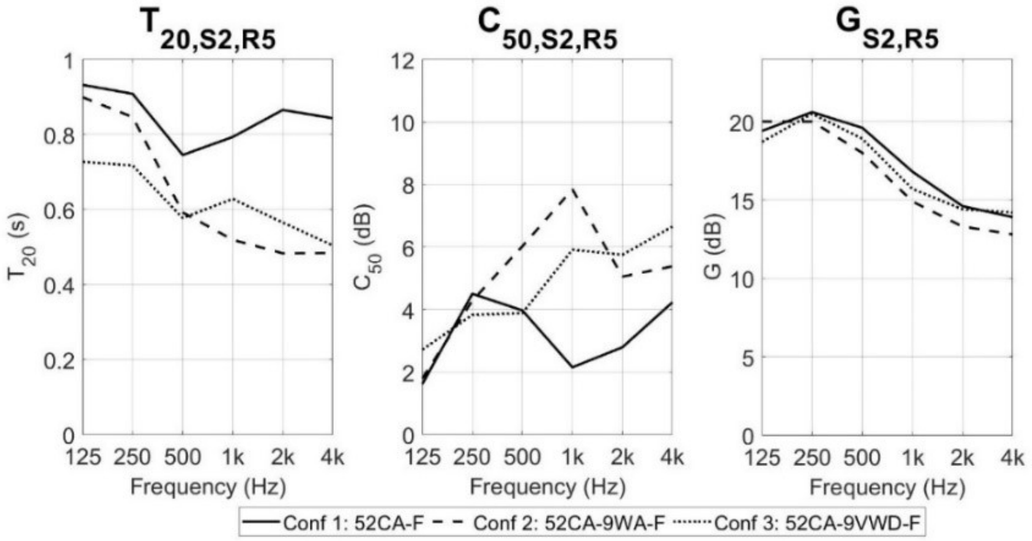

Figure S4. The three room acoustic parameters evaluated, reverberation time, T_{20}, speech clarity, C_{50}, and sound strength, G, for specific receiver positions using source position S2 and receiver position R5, i.e., same x-position as the speaker in the rear part of the room.
