# Supplementary material for: Subjective Experience of Speech Depending on the Acoustic Treatment in an Ordinary Room
Source: Int J Environ Res Public Health. 2021 Nov 23;18(23):12274. doi: 10.3390/ijerph182312274 (PMC8657266; doi:10.3390/ijerph182312274)
Supplement: Supplementary file 1 [file ijerph-18-12274-s001.zip › ijerph-1454273- Supplementary 2.pdf]

International Journal of
Environmental Research and Public Health

MDPI

Supplementary 2: Results on linear regression

Following two tables presents the linear regression to sound quality, **Table S2**, and attributes, **Table S3**, using room acoustic parameters as explanatory variable.

Table S2. Equations for linear regression with degree of explanation, R^2, for sound quality using room acoustic parameters as explanatory parameter.

**Room acoustic parameter**	**Frequency (Hz)**	Regression	
		Equation	R^2 (%)
T_{20}	125	7.03 – 0.168 x T20_125	0
T_{20}	250	12.56 - 7.17 x T20_250	11
T_{20}	500	14.00 – 11.28 x T20_500	44
T_{20}	1000	10.83 – 6.14 x T20_1000	35
T_{20}	2000	9.50 – 4.16 x T20_2000	30
T_{20}	4000	9.75 – 4.82 x T20_4000	34
C_{50}	125	6.38+0.32 x C50_125	28
C_{50}	250	5.82 + 0.289 x C50_250	30
C_{50}	500	3.65 + 0.659 x C50_500	24
C_{50}	1000	3.89 + 0.486 x C50_1000	68
C_{50}	2000	3.66 + 0.569 x C50_2000	85
C_{50}	4000	3.49 + 0.492 x C50_4000	67
G	125	3.28 + 0.19 x G_125	7
G	250	41.27 – 1.69 x G_250	28
G	500	9.46 – 0.13 x G_500	1
G	1000	4.13 + 0.16 x G_1000	3
G	2000	2.83 + 0.27 x G_2000	9
G	4000	1.42 + 0.37 x G_4000	11

Table S3. Equations for linear regression with degree of explanation, R^2, for attributes using room acoustic parameters as explanatory variable.

**Room acoustic parameter**	**Frequency (Hz)**	Regression	
		Equation	R^2
T_{20}	125	4.28 – 2.31 x T20_125	11
T_{20}	250	7.82 – 6.9 x T20_250	26
T_{20}	500	8.30 – 9.47 x T20_500	77
T_{20}	1000	6.22 – 6.06 x T20_1000	84
T_{20}	2000	5.10 – 4.41 x T20_2000	82
T_{20}	4000	5.10 – 4.67 x T20_4000	80
C_{50}	125	2.18 + 0.09 x C50_125	6
C_{50}	250	2.12 + 0.07 x C50_250	3
C_{50}	500	0.06 + 0.46 x C50_500	30
C_{50}	1000	0.46 + 0.30 x C50_1000	66
C_{50}	2000	0.76 + 0.288 x C50_2000	50
C_{50}	4000	0.31 + 0.29 x C50_4000	59
G	125	-0.98 + 0.17 x G_125	14
G	250	29.78 – 1.35 x G_250	43
G	500	11.21 – 0.46 x G_500	16
G	1000	2.78 – 0.03 x G_1000	0
G	2000	1.58 + 0.05 x G_2000	1
G	4000	1.57 + 0.05 x G_4000	1
